# Seroprevalence of Toscana and sandfly fever Sicilian viruses in humans and livestock animals from western Saudi Arabia

**DOI:** 10.1016/j.onehlt.2023.100601

**Published:** 2023-07-11

**Authors:** Sarah Ayman Al-numaani, Alaa Talat Al-Nemari, Sherif A. El-Kafrawy, Ahmed M. Hassan, Ahmed M. Tolah, Maimonah Alghanmi, Ayat Zawawi, Badr Essa Masri, Salwa I. Hindawi, Thamir A. Alandijany, Leena H. Bajrai, Abdullah Bukhari, Ahmad Bakur Mahmoud, Waleed S. Al Salem, Abdullah Algaissi, Remi N. Charrel, Esam I. Azhar, Anwar M. Hashem

**Affiliations:** a Vaccines and Immunotherapy Unit, King Fahd Medical Research Center, King Abdulaziz University, Jeddah, Saudi Arabia; b Department of Clinical Microbiology and Immunology, Faculty of Medicine, King Abdulaziz University, Jeddah, Saudi Arabia; c Special Infectious Agents Unit- BSL3, King Fahd Medical Research Center, King Abdulaziz University, Jeddah, Saudi Arabia; d Department of Medical Laboratory Sciences, Faculty of Applied Medical Sciences, King Abdulaziz University, Jeddah, Saudi Arabia; e Department of Medical Laboratory Technology, Faculty of Applied Medical Sciences, King Abdulaziz University, Rabigh, Saudi Arabia; f Department of Hematology, Faculty of Medicine, King Abdulaziz University, Jeddah, Saudi Arabia; g Biochemistry Department, Faculty of Sciences, King Abdulaziz University, Jeddah, Saudi Arabia.; h Department of Medicine, Faculty of Medicine, Imam Mohammed Ibn Saud Islamic University, Riyadh, Saudi Arabia; i Department of Medical Laboratory Technology, College of Applied Medical Sciences, Taibah University, Almadinah Almunwarah, Saudi Arabia; j Department of Agriculture, Ministry of Environment, Water and Agriculture, Riyadh, Saudi Arabia; k Department of Medical Laboratory Sciences, Faculty of Applied Medical Sciences, Jazan University, Jazan, Saudi Arabia; l Emerging and Epidemic Infectious Diseases Research Unit, Medical Research Center, Jazan University, Jazan, Saudi Arabia; m Unité des Virus Emergents (UVE: Aix Marseille Univ, IRD 190, INSERM 1207, IHU Méditerranée Infection), Marseille, France

**Keywords:** SFSV, TOSV, Livestock, Blood donors, Animal handlers

## Abstract

High seroprevalence rates of several phleboviruses have been reported in domestic animals and humans in sandfly-infested regions. Sandfly Fever Sicilian virus (SFSV) and Toscana virus (TOSV) are two of these viruses commonly transmitted by *Phlebotomus* sandflies. While SFSV can cause rapidly resolving mild febrile illness, TOSV could involve the central nervous system (CNS), causing diseases ranging from aseptic meningitis to meningoencephalitis. Sandfly-associated phleboviruses have not been investigated before in Saudi Arabia and are potential causes of infection given the prevalence of sandflies in the country. Here, we investigated the seroprevalence of SFSV and TOSV in the western region of Saudi Arabia in samples collected from blood donors, livestock animals, and animal handlers. An overall seroprevalence of 9.4% and 0.8% was found in humans for SFSV and TOSV, respectively. Seropositivity was significantly higher in non-Saudis compared to Saudis and increased significantly with age especially for SFSV. The highest seropositivity rate was among samples collected from animal handlers. Specifically, in blood donors, 6.4% and 0.7% tested positive for SFSV and TOSV nAbs, respectively. Animal handlers showed higher seroprevalence rates of 16% and 1% for anti-SFSV and anti-TOSV nAbs, respectively, suggesting that contact with livestock animals could be a risk factor. Indeed, sera from livestock animals showed seropositivity of 53.3% and 4.4% in cows, 27.5% and 7.8% in sheep, 2.2% and 0.0% in goats, and 10.0% and 2.3% in camels for SFSV and TOSV, respectively. Together, these results suggest that both SFSV and TOSV are circulating in the western region of Saudi Arabia in humans and livestock animals, albeit at different rates, and that age and contact with livestock animals could represent risk factors for infection with these viruses.

## Graphical abstract

**Figure:**
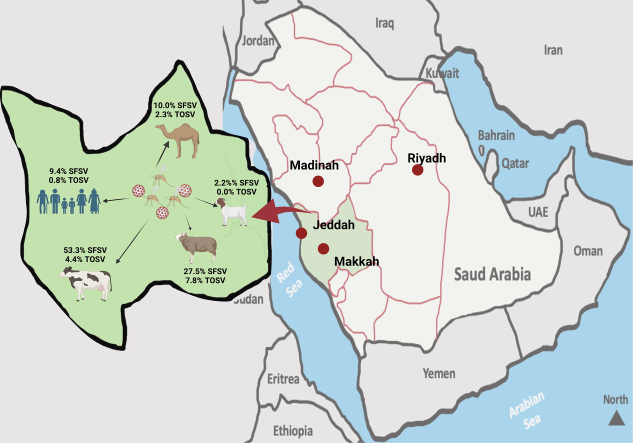

## Author summary

During the last decade, emerging viruses posed a constant threat to the world's public health. Sandfly-transmitted phleboviruses, including Sandfly Fever Sicilian virus (SFSV) and Toscana virus (TOSV), are endemic in many regions of the world; however, there is limited data about their circulation and related human infections in Saudi Arabia. Through a seroprevalence study, we studied the respective involvement of SFSV and TOSV in human and livestock animals in the western region of Saudi Arabia. Our data showed that (i) TOSV is present in the western region of Saudi Arabia but at a lower rate compared to SFSV, (ii) seropositivity was significantly higher in non-Saudis compared to Saudis and increased significantly with age, (iii) SFSV and TOSV are also widely distributed in different livestock animals including cows, sheep, goats and camels. Together, these results suggest that both SFSV and TOSV are circulating in the western region of Saudi Arabia in humans and livestock animals. Thus, further studies are needed to explore possible risk factors in the natural infection cycle; to define and quantify the veterinary importance of sandfly-transmitted phleboviruses. The results also highlight the extent of studying the role of SFSV and TOSV in humans and may lead to the set-up of diagnostic tests for blood donors and patients with unexplained febrile illness.

## 1 Introduction

Clinicians usually overlook sandfly-associated viruses such as phleboviruses (Bunyaviridae family), orbiviruses (Reoviridae family) and vesiculoviruses (Rhabdoviridae family) despite their suspected significance in human infections compared to other arboviruses such as flaviviruses and alphaviruses. This is mostly because of the insufficient epidemiological data and scarce availability of diagnostic tools which result in underestimation of the disease prevalence and often undermining the magnitude of the problem.

Sandfly-transmitted phleboviruses are common in many regions of the world including central Asia, the Middle East, the Indian subcontinent, the Mediterranean and Africa. They are transmitted by *Lutzomyia* and *Phlebotomus* sandflies in the New and the Old World, respectively [[1], [2], [3]].

Scientists classify Old World sandfly-borne phleboviruses into three major serological complexes based on their antigenic properties. These complexes include sandfly fever Naples serocomplex such as sandfly fever Naples virus (SFNV) and Toscana virus (TOSV), Salehabad serocomplex which includes viruses such as Salehabad virus (SALV) and Arbia virus, and sandfly fever Sicilian serocomplex which includes viral species such as sandfly fever Sicilian virus (SFSV) and Corfou virus (CFUV) [4]. Several other known and tentative viral species have also been identified and classified within these serocomplexes. Researchers have recently revisited the taxonomy of the Phlebovirus and classified 66 species within these serocomplexes [5]; however this does not affect antigenic relationships between viruses.

Several of these viruses are significant human pathogens causing a spectrum of symptoms ranging from a brief self-limiting febrile illness to encephalitis and meningoencephalitis. Viruses such as SFSV and SFNV mainly cause transient febrile disease commonly referred to as “sandfly fever” or “3-day fever” with an incubation period of 3–5 days, after which symptoms such as fever, malaise, myalgia, headache, photophobia and retro-orbital pain could last for 2–3 days [[6], [7], [8]]. While TOSV could also cause mild febrile illness with extended incubation period [9], severe clinical symptoms such as neurological manifestations and central and peripheral nervous system involvement are not uncommon [10,11]. Despite >50% of cases develop encephalitis or meningoencephalitis, and may require hospitalization in intensive care unit, the outcome of infection is usually favorable with little complications [[11], [12], [13]].

High seroprevalence rates of several phleboviruses have been reported in humans and animals in sandflies-infested regions. Except for reports on rift valley fever virus, a single seroepidemiological study was published from Saudi Arabia in 1976 [14] on the prevalence of antibodies against phleboviruses. The study tested 34 samples and showed a prevalence of 20.6% and 5.9% for SFSV and SFNV, respectively. The study did not show the sample collection sites or areas investigated in Saudi Arabia. However, this seminal study did not consider TOSV to be a potential human pathogen at that time.

Several species of sandflies infest Saudi Arabia and act as etiological vectors for phleboviruses associated such as *P. papatasi*, *P. sergenti*, *P, bergeroti*, *P. arabicus*, *P. tobbi*, *P. orientalis* and *P. alexandri* [15], together with other factors including climate change, city expansion and massive agricultural projects [16,17]. Therefore, several aspects remain to be investigated to increase our knowledge of phleboviruses and their vectors in the Arabian Peninsula.

**Fig. 1:**
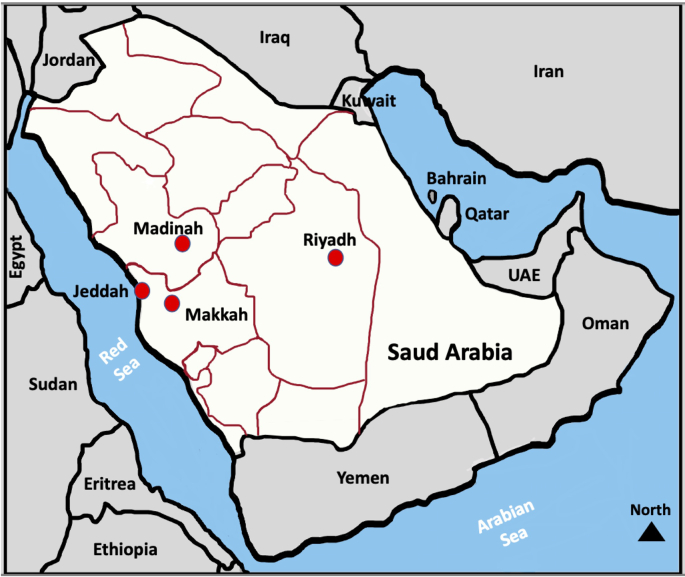
Map of Saudi Arabia shows site of sample collection from the city of Jeddah in the Western region of the country.

Currently, there is no data about these viruses and related human diseases in Saudi. Therefore, in the current study, we investigated the seroprevalence of SFSV and TOSV in samples collected between 2012 and 2019 from blood donors in western Saudi Arabia. In addition, we included samples from livestock animals and animal handlers who had been in contact with such animals. The team collected samples from the metropolitan city of Jeddah and neighbouring areas in the country's western region (Fig. 1). The city of Jeddah is the most populated city in the western region of the country and represents the main travel hub for tens of millions of Muslims from around the world who visit the holy places in Makkah and Madinah which host one of the largest global mass-gathering events annually.

This study represents a significant step towards our understanding of the epidemiology of SFSV and TOSV and establishing an effective surveillance system or improving the existing system. The study will also help to evaluate the potential risk factors associated with acquiring SFSV and TOSV infections in the western region of Saudi Arabia.

## 2 Material and method

### 2.1 Clinical samples

In this study, we used three different cohorts of archived serum samples collected from the city of Jeddah, Saudi Arabia. This included a total of 705 archived human serum samples collected between 2012 and 2016 from blood donors and 313 serum samples collected in 2019 from animal handlers (i.e. butchers and shepherds) who were in direct contact with livestock animals. Additionally, a total of 228 archived serum samples collected in 2019 from livestock animals (45 cows, 44 goats, 88 camels and 51 sheep); all samples were stored at −80 °C till tested. All samples were screened for SFSV and TOSV by live virus microneutralization assay. The study was approved by the Biomedical bioethics committee at the center of excellence in genomic medicine research (CEGMR), King Abdulaziz University Hospital (13-CEGMR-Bioeth-2020).

### 2.2 Cells and viruses

African Green monkey kidney-derived Vero E6 cells were cultured and maintained in Dulbecco's modification of Eagle medium (DMEM) with 10% heat-inactivated fetal bovine serum (FBS), 10 mM HEPES buffer (pH 7.2), 1× l-glutamine, 1% penicillin/streptomycin, and 2.2 g/L sodium bicarbonate (pH 7.2) at 37 °C in a humidified 5–7% CO_2_ incubator. Viral isolates TOSV (strain UVE/TOSV/2013/DZ/189 isolated from sandflies collected in Algeria [provided by the European Virus Archive catalog *https://www.european-virus-archive.com/virus/toscana-virus-strain-uvetosv2013dz189*] [18] and SFSV (strain UVE/SFSV/1943/IT/Sabin isolated from a human case that occurred in the Palermo region (Italy) during World War II (provided by the European Virus Archive catalog *https://www.european-virus-archive.com/virus/sandfly-fever-sicilian-virus-strain-uvesfsv1943itsabin*), provided by RNC, were amplified, titrated by standard TCID_50_ assay, and subsequently used in microneutralization assay in Vero E6 cells.

### 2.3 Viral microneutralization assays

Serum samples were initially screened for the presence of neutralizing antibodies (nAb) at 1:10 dilution. Heat-inactivated sera were diluted at 1:10 dilution in DMEM and co-incubated with an equal volume of DMEM containing 200 TCID_50_ of either TOSV or SFSV in a 96-well plate which was kept in a 5% CO_2_ incubator at 37 °C for 1 h. Then, 100 μL of Vero E6 suspension containing 2 × 10^5^ cells was added to all wells, and the plate was incubated at 37 °C in a 5% CO_2_ incubator for 5 days. Then, we examined the plates for the presence of cytopathic effect (CPE). Each serum sample was tested in duplicate. Virus only and cell only controls were included in each run-in duplicates as well. Positive rabbit sera containing neutralizing antibodies were raised against TOSV and SFSV and used as positive control sera. Positive samples were then used to determine the nAb titer by testing a two-fold serial dilution of each serum sample starting from 1:10 dilution. Titer was determined as the reciprocal of the highest dilution that protected cells from CPE in 50% of the wells (MNT_50_). The cutoff value for positivity was set at titer ≥10.

### 2.4 Statistical analysis

We reported categorical data as frequency and percentage (%) or Fisher's exact tests to compare percentages of positivity among categories of the same independent variables using the statistical package SPSS Version 21. We calculated confidence intervals (95% CI) for proportions by the Wilson method. A *p*-value of <0.05 was considered statistically significant.

## 3 Results

### 3.1 Demographics of human participants

We examined the seroprevalence of the SFSV and TOSV in both humans and animals in the western region of Saudi Arabia (Fig. 1). This study surveyed 1018 human participants, 705 of whom were blood donors and 313 of whom were animal handlers. The animal handlers were in continuous contact with livestock animals. Out of the total samples, 97.2% were from males compared to 2.8% from female subjects (Table 1). The majority of the samples were from non-Saudi individuals (66.7%). Most of the participants were in the age group from 31 to 50 years (51.3%), followed by individuals aged 30 years old or younger (45.3%). The mean age of the participants was 33 ± 8.9 years with a median age of 32 years and a range from 14 to 76 years.

**Table 1:** Overall human sample characteristics.

Characteristics	Overall	Blood donors	Animal handlers
	n (%)	n (%)	n (%)
**Gender**			
Female	28 (2.8)	28 (4.0)	0 (0.0)
Male	990 (97.2)	677 (96.0)	313 (100.0)
**Nationality**			
Saudi	339 (33.3)	338 (47.9)	1 (0.3)
Non-Saudi	679 (66.7)	367 (52.1)	312 (99.7)
**Age**			
≤ 30	461 (45.3)	351 (49.8)	110 (35.1)
31–50	522 (51.3)	342 (48.5)	180 (57.5)
> 51	35 (3.4)	12 (1.7)	23 (7.4)
**Total**	1018	705	313

While all 313 individuals from the animal handlers' group were males, 4% (*n* = 28) of blood donors were females compared to 96% males (*n* = 677). All except for 1 participant from the animal handlers' group were non-Saudis (99.7%). On the other hand, the percentage of Saudi and non-Saudi blood donors was comparable (47.9% vs 52.1%, respectively). As shown in Table 1, individuals aged 30 years old or younger and those in the age group 31–50 years were almost similar among the blood donors (49.8% and 48.5%, respectively). On the contrary, most participants from the animal handlers' group were between the age of 31–50 years (57.5%), followed by those 30 years old or younger (35.1%). The mean age among the animal handlers' group was 36 ± 9.9 years with a median of 35 years, and a range from 14 to 76 years, which was greater than the mean age of the blood donors which was 31.65 ± 8 years with a median of 31 years and a range from 17 to 67 years.

### 3.2 Seroprevalence of SFSV and TOSV

The overall seroprevalence for SFSV and TOSV in humans was 9.4% and 0.8%, respectively. Seropositivity was only found in males and significantly higher in non-Saudis compared to Saudis (Table 2). Specifically, we found that 10.9% and 5.6% of non-Saudis and Saudis were seropositive for SFSV, respectively. Similarly, seropositivity for TOSV was only found in non-Saudis (1.2%) as shown in Table 2. Interestingly, seroprevalence for both viruses increased significantly with age, especially for SFSV. None of the samples was found positive for both viruses.

**Table 2:** Overall seroprevalence of SFSV and TOSV in humans.

		SFSV	TOSV
Characteristics	Tested	n (%; 95% CI)	n (%; 95% CI)
**Gender**			
Female	28	0 (0.0; 0.0–12.1)	0 (0.0; 0.0–12.1)
Male	990	93 (9.4; 7.7–11.4)	8 (0.8; 0.4–1.6)
**Nationality**⁎			
Saudi	339	19 (5.6; 3.6–8.6)	0 (0.0; 0.0–1.1)
Non-Saudi	679	74 (10.9; 8.8–13.5)	8 (1.2; 0.6–2.3)
**Age**⁎			
≤ 30	461	33 (7.2; 5.1–9.9)	3 (0.7; 0.2–1.9)
31–50	522	51 (9.8; 7.5–12.6)	4 (0.8; 0.3–2.0)
> 51	35	9 (25.7; 14.2–42.1)	1 (2.9; 0.5–14.5)
**Total**	1018	93 (9.1; 7.5–11.1)	8 (0.8; 0.4–1.5)

⁎ Statistically significant (*p* < 0.001) in SFSV but not TOSV. The cut-off value for positivity was set at titer ≥10.

**Fig. 2:**
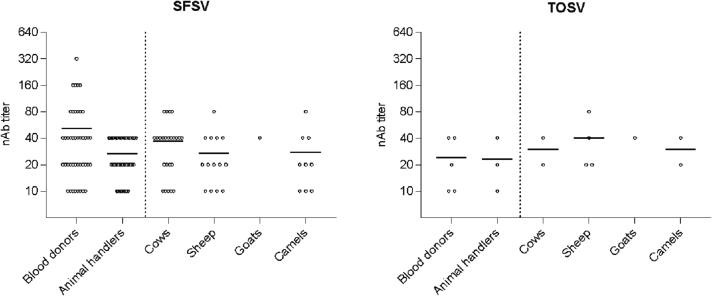
Neutralizing antibodies (nAb) titers in humans and animals against SFSV and TOSV.

In the blood donors, 43 samples (6.4%) tested positive for SFSV and 5 samples (0.7%) tested positive for TOSV nAbs (Table 3). Among animal handlers, higher percentages of 16% and 1% of the samples were positive for anti-SFSV and anti-TOSV nAbs, respectively, suggesting that contact with livestock animals could be a risk factor for infection with these viruses (Table 3). Table 3 shows that non-Saudis had a higher seropositivity rate than Saudis, except for SFSV in blood donors, where the seroprevalence rate was similar in both groups. As shown in Fig. 2, the nAb titers for TOSV ranged between 10 and 40 in both groups. On the other hand, nAb titers for SFSV were statistically higher in healthy blood donor and ranged from (10−320) compared to the animal handlers' group (10–40).

**Table 3:** Seroprevalence of SFSV and TOSV in blood donors and animal handlers.

	Blood donors			Animal handlers		
		SFSV	TOSV		SFSV	TOSV
**Characteristics**	**Tested**	**n (%; 95% CI)**	**n (%; 95% CI)**	**Tested**	**n (%; 95% CI)**	**n (%; 95% CI)**
**Gender**						
Female	28	0 (0.0; 0.0–12.1)	0 (0.0; 0.0–12.1)	0	0	0
Male	677	43 (6.4; 4.7–8.4)	5 (0.7; 0.3–1.7)	313	50 (16.0; 12.3–20.4)	3 (1.0; 0.3–2.8)
**Nationality**						
Saud	338	19 (5.6; 3.6–8.6)	0 (0.0; 0.0–1.1)	1	0 (0.0; 0.0–79.3)	0 (0.0; 0.0–79.3)
Non-Saudi	367	24 (6.5; 4.4–9.5)	5 (1.4; 0.6–3.2)	312	50 (16.0; 2.4–20.5)	3 (1.0; 0.3–2.8)
**Age**⁎						
≤ 30	351	20 (5.7; 3.7–8.6)	2 (0.6; 0.2–2.1)	110	13 (11.8; 7.0–19.2)	1 (0.9; 0.2–5.0)
31–50	342	22 (6.4; 4.3–9.5)	3 (0.9; 0.3–2.5)	180	29 (16.1; 11.5–22.2)	1 (0.6; 0.1–3.1)
> 51	12	1 (8.3; 1.5–35.4)	0 (0.0; 0.0–24.3)	23	8 (34.8; 18.8–55.1)	1 (4.3; 0.8–21.0)
**Total**	705	43 (6.1; 4.6–8.1)	5 (0.7; 0.3–1.6)	313	50 (16.0; 12.3–20.4)	3 (1.0%)

⁎ Statistically significant (p < 0.001) in SFSV but not TOSV in animal handlers' group but not blood donors. The cut-off value for positivity was set at titer ≥10.

The higher seroprevalence in animal handlers suggested potential circulation of SFSV and TOSV in the vicinity of livestock animals. Therefore, we tested 228 serum samples obtained from livestock animals, including cows (45 samples), sheep (51 samples), camels (88 samples) and goats (44 samples). Seroprevalence for SFSV was higher than TOSV in these animals in concordance with the rates found in humans (Table 4). Cows showed the highest seropositivity for SFSV (53.3%) while sheep had the highest seroprevalence for TOSV (7.8%). Goats showed the lowest seropositivity for both viruses. Titers ranged between 10 and 80 as shown in Fig. 1.

**Table 4:** Seroprevalence in livestock animals.

Animal	Tested	SFSV	TOSV
		n (%)	n (%)
Cows	45	24 (53.3)	2 (4.4)
Sheep	51	14 (27.5)	4 (7.8)
Goats	44	1 (2.2)	0 (0.0)
Camels	88	9 (10.0)	2 (2.3)

Together, these results suggest that both SFSV and TOSV are circulating in the western region of Saudi Arabia in humans and livestock animals, albite at different rates, and that age and contact with livestock animals could represent risk factors for infection with these viruses.

## 4 Discussion

Both SFSV and TOSV are phleboviruses (family *Phenuiviridae*, order *Bunyavirales*). Both viruses are considered endemic in the Middle East and Mediterranean area where they are transmitted to humans by infected sandflies [14,19]. Most human infections by these viruses are asymptomatic but infection can lead to an acute febrile illness that lasts for 3 to 5 days with fever, myalgia, and headaches [20]. However, TOSV has a marked tropism for the CNS, as the only known sandfly-borne phlebovirus to cause neurological infection and is regarded as an important cause of acute aseptic meningitis and meningoencephalitis [[21], [22], [23]]. Since infection by these viruses can be asymptomatic, the actual prevalence of infection is likely underreported although several seroprevalence studies confirmed the widespread nature of SFSV and TOSV infection in different geographical zones like Southern Europe, the Mediterranean region, Africa, West Asia, and the Middle East [14,20,[24], [25], [26], [27]].

In Saudi Arabia, which is an Afrotropical country in which vector-borne diseases are considered an emerging threat, there is no data about these viruses and related human diseases. Furthermore, no population-wide serological studies were reported in Saudi Arabia although previous inventory studies of the sandfly species in Saudi Arabia indicated that the species described as vectors for either SFSV or TOSV are present in the kingdom [28]. Studies have identified the most important *Phlebotomus spp* known as medically important sandfly in addition to non-medically important *Sergentomyia* spp. Mostly, *P. papatasi* was found to be predominant in very arid areas while *P. bergroti* and *P. sergenti* were more likely to be in higher altitude regions [29]. Doha et al. reported the inventory of phlebotomine sandflies between 1996 and 1997 in Al-Baha province (southwestern Saudi Arabia) [30]. They reported *P. bergeroti* (41.7%) as the predominant species, followed by *S. antennata, S. tiberiadis, P. sergenti*, *P. papatasi* and *P. arabicus* (each ranging between 9.6 and 11%), and *P. alexandri*, *P. orientalis* and *S. clydei* (ranging between 1.1 and 3%). A similar study in Asir region (southwestern Saudi Arabia) reported *P. bergeroti* (85.7%) followed by *P. sergenti* (11%) and other species including *P. alexandri*, *P. papatasi*, *P. orientali* and *P. arabicus* (<1%) [31]. Another epidemiological survey conducted in the city of Hail (northwestern Saudi Arabia) in 2015–2016 found *P. papatasi*, *P. kazeruni* and *S. clydei* as dominant species [32]. In Al-Madinah (Northeast Saudi Arabia), a study was conducted to inventory sandflies where cutaneous leishmaniasis is documented [33]; *P. papatasi* was the most predominant species which encompassed >70% of the sandfly population. Similar studies have shown *P. papatasi* (99%) to be the most prevalent species followed by *P. sergenti* (1%) in the city of Riyadh [34]. However, all these studies have mainly investigated leishmaniasis but not the circulation of sandfly-borne viruses. Leishmaniasis, specifically cutaneous leishmaniasis, is endemic in Saudi Arabia. According to the literature, in addition to the presence of the etiological sandfly vector, factors like continuous migration, population movement, climate change, massive agricultural projects and rapid urban and cities expansion represent major risk factors that could increase the transmission of leishmania and other sandfly-borne viruses to humans and animals [35].

In the present study, we have attempted to perform a large-scale population-wide study to understand the prevalence of SFSV and TOSV infections in Jeddah as a model city for the western region of Saudi Arabia as Jeddah represent the main travel hub city for tens of millions of pilgrims coming to visit the Holy places in Makkah and Medinah. Using viral microneutralization assay, we investigated the seroprevalence of SFSV and TOSV in samples collected between 2012 and 2019 from blood donors, animal handlers and from livestock animals in the western region of Saudi Arabia. Of note, while one cannot completely exclude the presence of cross-reactive nAbs especially at low titers, viral microneutralization assay is considered the most currently available stringent and specific serological assay for phleboviruses [[36], [37], [38], [39]].

Overall, our results showed a seroprevalence of 9.4% and 0.8% for SFSV and TOSV, respectively. The highest seropositivity rate was among samples collected from animal handlers; 16% tested positive for SFSV and 1% for TOSV. These data indicate a potentially high prevalence of SFSV and TOSV in the western region of Saudi Arabia and that exposure to animals increases the risk of both SFSV and TOSV infections. We found seropositivity only in adult males and it was significantly higher in non-Saudis compared to Saudis. While there is a gender and nationality bias in our study, this bias reflects the actual demographics of the sampled populations in Saudi. This is mostly due to socioeconomic factors as most people in Saudi Arabia who work in animal slaughterhouses and animal husbandry are foreigner males. Furthermore, most blood donors in Saudi are males. We also observed an age-dependent increase in the seropositivity of both SFSV and TOSV in our study which was probably related to a cumulative exposure of older males to these viruses via the more frequent interaction with infected animals or sandflies vectors. Compared to the only report on SFSV in Saudi Arabia that showed a seroprevalence of about 20.6%, our data showed lower rates [14]. In addition to the small sample size in the previous study, it is unclear whether samples collected were collected from healthy or high-risk individuals or from non-locals who visited the country temporarily compared to the large sample size in our study, which shows higher prevalence in non-Saudis [14]. Although the only known mode of transmission of these viruses is through the bite of a sandfly, the seropositivity of blood donors for these viruses raise concerns about the theoretical risks of virus transmission through blood transfusion and organ transplants [40], which is worth investigating in future studies.

Livestock like cows, sheep, goats and camels can be infected by such viruses. Indeed, a previous study showed that >50% of the cattle were infected with SFSV, while 22% of goats were found to be SFSV positive [41]. In another study, animals like dogs and cats were found to be positive for both SFSV and TOSV [37]. However, the exact pathogenic role of these viruses in livestock is not yet fully understood [36]. Therefore, we also tested the serum samples obtained from cows, sheep, goats and camels. Our study found that the highest number of SFSV positive samples were found in cows. Out of 45 samples, 24 samples (53.0%) tested positive for anti-SFSV Abs, followed by sheep (27.5%), camels (10%) and goats which showed the lowest positivity (2.2%). TOSV seroprevalence in livestock animals observed in our study was lower than that for SFSV. We found seropositivity of 7.8% in sheep, 4.4% in cows, 2.3% in camels and 0.0% in goats. While these rates of TOSV in livestock animals were lower than those reported by others in Spain, for example, our data were close to rates reported in other studies, suggesting that seroprevalence of these viruses could vary significantly between regions [38,39]. While these data strongly suggest the widespread of these viruses in livestock animals, researchers should be deliberate on the possible role of livestock as reservoirs or dead-end hosts for these viruses, as it is yet not fully understood and require further studies [42].

The main limitation of the current study was the inability to further confirm the seropositivity of the samples using viral isolation or molecular techniques such as next-generation sequencing which could have helped identifying other potential unknown viruses. Furthermore, no clinical data were collected nor available on the participants, so it was not possible to identify acute cases. Although cross-reactivity in viral microneutralization is expected to be low and we have not found any sample that is positive for both viruses, cross- reactivity at low titers is still possible. Finally, there is a gender and nationality bias in our study which is due to the actual demographics of the sampled populations in Saudi.

In conclusion, this is the first large-scale population-based seroepidemiological analysis to measure the prevalence of SFSV and TOSV in Saudi Arabia. Together, our data suggest that both SFSV and TOSV are circulating in the western region of Saudi Arabia in humans and livestock animals, albeit at different rates, and that working in the vicinity of livestock animals could represent a risk factor. Yet, several aspects remain to be investigated to increase our knowledge of phleboviruses biology, their vectors, mammalian reservoirs, epidemiology and associated diseases in humans and animals. Furthermore, there is a huge gap in our knowledge about the potential risk factors that could increase the transmission of sandfly-borne pathogens such as continuous migration, population movement, climate change, massive agricultural projects and rapid urban and cities expansion. Therefore, further investigations are needed not only to better assess such risks but also to develop diagnostics, isolate and identify strains of SFSV and TOSV as well as other phleboviruses from animals, humans and sandflies, determine the potential risk associated with blood transfusion and help enhancing the awareness among those at higher exposure risk.

## Author contributions

SAA and ATA contributed equally to this work. RNC, EIA and AMH conceptualized and supervised the work, and contributed to the experimental design and analyses. SIH, TAA, LHB, AB, ABM, WSA, AAlgaissi, RNC, EIA and AMH collected samples and provided reagents and materials. SAA, ATA, SAE, AMHassan, AMT and BM performed and optimized experiments. SAA, ATA, SAE, MA, AA and AMH analyzed the data. RNC and AMH obtained funding. SAA, ATA, MA, AZ, AA and AMH drafted the manuscript. MA, AA WSA, RNC, EIA and AMH revised and edited manuscript drafts. All authors reviewed and approved the manuscript.

## Funding

This project was funded by the Deanship of scientific research (DSR), King Abdualziz University (KAU), Jeddah, under grant no. (G:1339-140-1440). The authors, therefore, acknowledge with thanks DSR for technical and financial support. This work was partly supported by the 10.13039/501100000780European Commission (European Virus Archive Global project (EVA GLOBAL, grant agreement No 871029) of the Horizon 2020 research and innovation programme) and virus strains were provided by the European virus archive-Marseille (EVAM) under the label technological platforms of Aix-Marseille.

## Ethics approval and consent to participate

The study was approved by the Biomedical bioethics committee at the center of excellence in genomic medicine research (CEGMR), King Abdulaziz University Hospital (13-CEGMR-Bioeth-2020).

## Consent for publication

Not applicable.

## Declaration of Competing Interest

The authors declare that they have no competing interests.

## Data Availability

Data will be made available on request.

## References

[1] Killick-Kendrick R. (1999). The biology and control of phlebotomine sand flies. Clin. Dermatol..

[2] Tesh R.B. (1988). The genus Phlebovirus and its vectors. Annu. Rev. Entomol..

[3] Weaver S.C., Barrett A.D. (2004). Transmission cycles, host range, evolution and emergence of arboviral disease. Nat. Rev. Microbiol..

[4] Ayhan N., Charrel R.N. (2018). Emergent Sand Fly–Borne Phleboviruses in the Balkan Region. Emerg. Infect. Dis..

[5] Kuhn J.H., Adkins S., Agwanda B.R., Al Kubrusli R., Alkhovsky S.V., Amarasinghe G.K. (2021). Correction to: 2021 taxonomic update of phylum Negarnaviricota (Riboviria: Orthornavirae), including the large orders Bunyavirales and Mononegavirales. Arch. Virol..

[6] Sabin A.B. (1951). Experimental studies on Phlebotomus (pappataci, sandfly) fever during World War II. Arch Gesamte Virusforsch..

[7] Bartelloni P.J., Tesh R.B. (1976). Clinical and serologic responses of volunteers infected with phlebotomus fever virus (Sicilian type). Am. J. Trop. Med. Hyg..

[8] Alkan C., Bichaud L., de Lamballerie X., Alten B., Gould E.A., Charrel R.N. (2013). Sandfly-borne phleboviruses of Eurasia and Africa: epidemiology, genetic diversity, geographic range, control measures. Antivir. Res..

[9] Laroche L., Jourdain F., Ayhan N., Bañuls A.L., Charrel R., Prudhomme J. (2021). Incubation period for neuroinvasive toscana virus infections. Emerg. Infect. Dis..

[10] Ayhan N., Charrel R.N. (2020). An update on Toscana virus distribution, genetics, medical and diagnostic aspects. Clin. Microbiol. Infect..

[11] Charrel R.N., Gallian P., Navarro-Mari J.M., Nicoletti L., Papa A., Sánchez-Seco M.P. (2005). Emergence of Toscana virus in Europe. Emerg. Infect. Dis..

[12] Dionisio D., Esperti F., Vivarelli A., Valassina M. (2003). Epidemiological, clinical and laboratory aspects of sandfly fever. Curr. Opin. Infect. Dis..

[13] Calamusa G., Valenti R.M., Vitale F., Mammina C., Romano N., Goedert J.J. (2012). Seroprevalence of and risk factors for Toscana and Sicilian virus infection in a sample population of Sicily (Italy). J. Inf. Secur..

[14] Tesh R.B., Saidi S., Gajdamovic S.J., Rodhain F., Vesenjak-Hirjan J. (1976). Serological studies on the epidemiology of sandfly fever in the Old World. Bull. World Health Organ..

[15] Al-Salem W.S., Solórzano C., Weedall G.D., Dyer N.A., Kelly-Hope L., Casas-Sánchez A. (2019). Old World cutaneous leishmaniasis treatment response varies depending on parasite species, geographical location and development of secondary infection. Parasit. Vectors.

[16] Kassem H.A., El Nogoumy N.N., El Sawaf B.M. (2012). Impact of urbanization on the sand fly Phlebotomus langeroni nitzulescu in an old focus of visceral leishmaniasis in Egypt. J. Egypt. Soc. Parasitol..

[17] Alten B., Maia C., Afonso M.O., Campino L., Jiménez M., González E. (2016). Seasonal dynamics of phlebotomine sand Fly species proven vectors of mediterranean leishmaniasis caused by leishmania infantum. PLoS Negl. Trop. Dis..

[18] Alkan C., Allal-Ikhlef A.B., Alwassouf S., Baklouti A., Piorkowski G., de Lamballerie X. (2015). Virus isolation, genetic characterization and seroprevalence of Toscana virus in Algeria. Clin. Microbiol. Infect..

[19] Karabatsos N. (1978). Supplement to international catalogue of arboviruses including certain other viruses of vertebrates. Am. J. Trop. Med. Hyg..

[20] Alkan C., Moin Vaziri V., Ayhan N., Badakhshan M., Bichaud L., Rahbarian N. (2017). Isolation and sequencing of Dashli virus, a novel Sicilian-like virus in sandflies from Iran; genetic and phylogenetic evidence for the creation of one novel species within the Phlebovirus genus in the Phenuiviridae family. PLoS Negl. Trop. Dis..

[21] Cusi M.G., Savellini G.G. (2011). Diagnostic tools for Toscana virus infection. Expert Rev. Anti-Infect. Ther..

[22] Doudier B., Ninove L., Million M., de Lamballerie X., Charrel R.N., Brouqui P. (2011). Unusual Toscana virus encephalitis in southern France. Med. Mal. Infect..

[23] Kuhn J.H., Adkins S., Agwanda B.R., Al Kubrusli R., Alkhovsky S.V., Amarasinghe G.K. (2021). 2021 taxonomic update of phylum Negarnaviricota (Riboviria: Orthornavirae), including the large orders Bunyavirales and Mononegavirales. Arch. Virol..

[24] McCarthy M.C., Haberberger R.L., Salib A.W., Soliman B.A., El-Tigani A., Khalid I.O. (1996). Evaluation of arthropod-borne viruses and other infectious disease pathogens as the causes of febrile illnesses in the Khartoum Province of Sudan. J. Med. Virol..

[25] Cohen D., Zaide Y., Karasenty E., Schwarz M., LeDuc J.W., Slepon R. (1999). Prevalence of antibodies to West Nile fever, sandfly fever Sicilian, and sandfly fever Naples viruses in healthy adults in Israel. Public Health Rev..

[26] Batieha A., Saliba E.K., Graham R., Mohareb E., Hijazi Y., Wijeyaratne P. (2000). Seroprevalence of West Nile, Rift Valley, and sandfly arboviruses in Hashimiah, Jordan. Emerg. Infect. Dis..

[27] Maroli M., Feliciangeli M.D., Bichaud L., Charrel R.N., Gradoni L. (2013). Phlebotomine sandflies and the spreading of leishmaniases and other diseases of public health concern. Med. Vet. Entomol..

[28] Ayhan N., Prudhomme J., Laroche L., Bañuls A.L., Charrel R.N. (2020). Broader geographical distribution of toscana virus in the mediterranean region suggests the existence of larger varieties of sand fly vectors. Microorganisms..

[29] Mondragon-Shem K., Al-Salem W.S., Kelly-Hope L., Abdeladhim M., Al-Zahrani M.H., Valenzuela J.G. (2015). Severity of old world cutaneous leishmaniasis is influenced by previous exposure to sandfly bites in Saudi Arabia. PLoS Negl. Trop. Dis..

[30] Doha S.A., Samy A.M. (2010). Bionomics of phlebotomine sand flies (Diptera: Psychodidae) in the province of Al-Baha, Saudi Arabia. Mem. Inst. Oswaldo Cruz.

[31] Ibrahim A.A., Abdoon M.A. (2005). Distribution and population dynamics of Phlebotomus sand flies (Diptera: Psychodidae) in an endemic area of cutaneous leishmaniasis in Asir Region, Southwestern Saudi Arabia. J. Entomol..

[32] Haouas N., Amer O., Alshammri F.F., Al-Shammari S., Remadi L., Ashankyty I. (2017). Cutaneous leishmaniasis in northwestern Saudi Arabia: identification of sand fly fauna and parasites. Parasit. Vectors.

[33] El-Badry A., Al-Juhani A., El-K Ibrahim, Al-Zubiany S. (2008). Distribution of sand flies in El-Nekheil province, in Al-Madinah Al-Munawwarah region, western of Saudi Arabia. Parasitol. Res..

[34] Mustafa M.B., Hussein S.M., Ibrahim E.A., Al-Seghayer S.M., Al Amri S.A., Gradoni L. (1994). Phlebotomus papatasi (Scopoli), vector of zoonotic cutaneous leishmaniasis in Riyadh province, Saudi Arabia. Trans. R. Soc. Trop. Med. Hyg..

[35] Abuzaid A.A., Abdoon A.M., Aldahan M.A., Alzahrani A.G., Alhakeem R.F., Asiri A.M. (2017). Cutaneous leishmaniasis in Saudi Arabia: a comprehensive overview. Vector Borne Zoonotic Dis..

[36] Lelli D., Scanferla V., Moreno A., Sozzi E., Ravaioli V., Renzi M. (2021). Serological evidence of phleboviruses in domestic animals on the pre-Apennine Hills (northern Italy). Viruses..

[37] Alwassouf S., Maia C., Ayhan N., Coimbra M., Cristovao J.M., Richet H. (2016). Neutralization-based seroprevalence of Toscana virus and sandfly fever Sicilian virus in dogs and cats from Portugal. J. Gen. Virol..

[38] Navarro-Marí J.M., Palop-Borrás B., Pérez-Ruiz M., Sanbonmatsu-Gámez S. (2011). Serosurvey study of Toscana virus in domestic animals, Granada, Spain. Vector Borne Zoonotic Dis..

[39] Ayhan N., Sherifi K., Taraku A., Bërxholi K., Charrel R.N. (2017). High rates of neutralizing antibodies to toscana and sandfly fever sicilian viruses in livestock, Kosovo. Emerg. Infect. Dis..

[40] Brisbarre N., Attoui H., Gallian P., Di Bonito P., Giorgi C., Cantaloube J.F. (2011). Seroprevalence of Toscana virus in blood donors, France, 2007. Emerg. Infect. Dis..

[41] Ayhan N., Charrel R.N. (2017). Of phlebotomines (sandflies) and viruses: a comprehensive perspective on a complex situation. Curr. Opin. Insect. Sci..

[42] Dincer E., Gargari S., Ozkul A., Ergunay K. (2015). Potential animal reservoirs of Toscana virus and coinfections with Leishmania infantum in Turkey. Am. J. Trop. Med. Hyg..

